# A comparison of clofarabine with ara-C, each in combination with daunorubicin as induction treatment in older patients with acute myeloid leukaemia

**DOI:** 10.1038/leu.2016.225

**Published:** 2016-09-30

**Authors:** A K Burnett, N H Russell, R K Hills, J Kell, O J Nielsen, M Dennis, P Cahalin, C Pocock, S Ali, S Burns, S Freeman, D Milligan, R E Clark

**Affiliations:** 1 Department of Haematology, Cardiff University School of Medicine, Cardiff, UK; 2 Department of Haematology, Nottingham University Hospital NHS Trust, Nottingham, UK; 3 Centre for Trails Research, Cardiff University, Cardiff, UK; 4 Department of Haematology, University Hospital of Wales Cardiff, Cardiff, UK; 5 Department of Haematology, Rigshospitalet, Copenhagen, Denmark; 6 Department of Haematology, Christie Hospital, Manchester, UK; 7 Department of Haematology, Blackpool Victoria Hospital, Blackpool, UK; 8 Department of Haematology, Kent & Canterbury Hospital, Canterbury, Kent, UK; 9 Department of Haematology, Castle Hill Hospital, Hull, UK; 10 Department of Immunology, University of Birmingham, Birmingham, UK; 11 Department of Haematology, Heartlands Hospital, Birmingham, UK; 12 Department of Haematology, Royal Liverpool University Hospital, Liverpool, UK

## Abstract

The study was designed to compare clofarabine plus daunorubicin vs daunorubicin/ara-C in older patients with acute myeloid leukaemia (AML) or high-risk myelodysplastic syndrome (MDS). Eight hundred and six untreated patients in the UK NCRI AML16 trial with AML/high-risk MDS (median age, 67 years; range 56–84) and normal serum creatinine were randomised to two courses of induction chemotherapy with either daunorubicin/ara-C (DA) or daunorubicin/clofarabine (DClo). Patients were also included in additional randomisations; ± one dose of gemtuzumab ozogamicin in course 1; 2v3 courses and ± azacitidine maintenance. The primary end point was overall survival. The overall response rate was 69% (complete remission (CR) 60% CRi 9%), with no difference between DA (71%) and DClo (66%). There was no difference in 30-/60-day mortality or toxicity: significantly more supportive care was required in the DA arm even though platelet and neutrophil recovery was significantly slower with DClo. There were no differences in cumulative incidence of relapse (74% vs 68% hazard ratio (HR) 0.93 (0.77–1.14), *P*=0.5); survival from relapse (7% vs 9% HR 0.96 (0.77–1.19), *P*=0.7); relapse-free (31% vs 32% HR 1.02 (0.83–1.24), *P*=0.9) or overall survival (23% vs 22% HR 1.08 (0.93–1.26), *P*=0.3). Clofarabine 20 mg/m^2^ given for 5 days with daunorubicin is not superior to ara-C+daunorubicin as induction for older patients with AML/high-risk MDS.

## Introduction

Although progress has been made with intensive treatments for younger patients with acute myeloid leukaemia (AML),^1, 2^ improvement in older patients given the same schedules is much more modest.^3, 4^ This is usually attributed to the higher frequency of disease with adverse biological features than in younger patients, which responds less frequently and for shorter duration. The other difference is that co-morbidities accumulate with age, whether apparent or not, and the tolerance to intensive therapy is poorer with a higher risk of induction mortality and reduced ability to deliver the total planned treatment.^5, 6^ More effective treatments are therefore required, which are both more efficacious and also have better tolerability.

Clofarabine (2-chloro-2′-fluoro-deoxy-9-ß-d-arabinofuranosyladenine) is a novel nucleoside analogue which was developed in a large screening programme to find new therapeutics which incorporate the beneficial properties of this class of drugs. In particular, fludarabine and cladribine are active as single agents in AML but at dose levels associated with prohibitive toxicity, which is due to the cleavage product 2-fluoroadenine being converted to toxic 2-fluoroadenosine.^7, 8^ Clofarabine is the result of a programme of development exploring a series of chemical modifications to minimise cleavage while retaining activity.^9^ It depends on membrane nucleoside transporters for cell entry and is sequentially phosphorylated in deoxycytidine kinase-dependent steps to the triphosphate, the active form of which is retained within cells for longer than other purine nucleoside analogues. Following initial studies in relapsed disease which confirmed its activity,^10^ two reports incorporating three un-randomised studies^11, 12^ assessed the front-line activity using lower doses in older patients who were considered unfit for intensive therapy. These studies were consistent in delivering complete remission to more than 40% of patients, and of interest this responsiveness did not seem to be limited by age or cytogenetic risk group. Given its favourable toxicity profile, potential for oral administration and similarity to fludarabine, it became a potential candidate novel therapy for the older patient. These trials did not establish the duration of response. In one of these pilot trials no renal function restriction was required, hence a dose level of 20 mg/m^2^ was tested in a small number of patients against the conventional 30 mg/m^2^ dose and produced a similar efficacy, but with a more favourable toxicity profile.^11^

On this basis we conducted a randomised trial in patients not considered suitable for intensive therapy, comparing clofarabine at a daily dose of 20 mg/m^2^ for five consecutive days, with low-dose ara-C (20 mg b.i.d. for 10 days).^13^ The remission rate was doubled by clofarabine, but overall survival (OS) was not improved because the survival of patients who failed to achieve complete remission on the clofarabine arm was worse, and when patients relapsed from a clofarabine-induced remission their survival was poorer than for those who relapsed from low-dose ara-C. In the current study as part of the United Kingdom National Cancer Research Institute (NCRI) AML16 Trial (ISRCTN 11036523), we wished to test clofarabine combined with daunorubicin vs ara-C combined with daunorubicin as induction therapy for older patients for whom conventional therapy was indicated.

## Patients and methods

The protocol was designed for older patients, generally age >60 years, who did not have blast transformation of chronic myeloid leukemia or acute promyelocytic leukemia. Patients with high-risk myelodysplastic syndrome (MDS), which was defined as >10% marrow blasts at diagnosis, were eligible. A small number of patients aged <60 years, who were not considered suitable for the concurrent AML15 trial for younger patients (which included high-dose ara-C), were included. Secondary AML was defined as resulting from either antecedent haematologic disorder or prior chemotherapy treatment for a non-haematological malignancy. The trial was conducted in accordance with the Declaration of Helsinki; it was sponsored by Cardiff University and was approved by Wales REC 3. All patients provided written informed consent to random treatment assignment. Patients were randomly assigned between two courses of chemotherapy comprising daunorubicin/ara-C or daunorubicin/clofarabine. To be eligible for randomisation assignment, patients were required to have serum creatinine within local normal limits.

**Figure 1:**
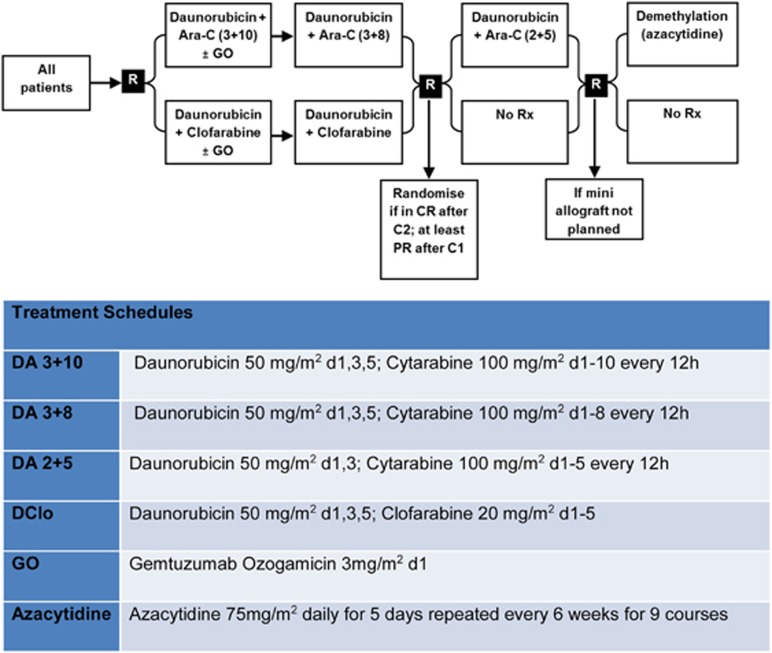
Trial design of AML16 (acute myeloid leukemia; intensive arm) from 2006 to 2009. C, course; CR, complete remission; PR, partial remission; Rx, treatment.

The treatment plan is set out in Figure 1 and the number of patients in the DClo vs DA induction randomisation who entered these randomisations is included in Table 1.

The median age of the patients was 67 years (range 56–84), 59% were male, 72% had *de novo* AML, 17% secondary AML and 11% had high-risk MDS. Six percent had a WHO performance score of >2; 14% had an *FLT3* mutation and 20% had an *NPM1c* mutation. The cytogenetic risk category as previously defined^14^ was 4% favourable, 73% intermediate and 23% adverse risk. Using the validated Wheatley Risk Score,^15^ which is based on age, cytogenetics, presenting white count, presence of secondary disease and performance status, three risk groups were defined (30% were good, 34% standard and 36% poor) with survival at 2 years of 29, 17 and 9%, respectively.

### Statistical methods

The primary outcome measure for the trial was OS. The study was powered to detect a difference of 10% in absolute 2-year survival from 25 to 35% (equivalent to hazard ratio (HR) of 0.76), with 90% power at *P*<0.05. This required a minimum of 552 deaths, and at least 800 patients had to be recruited. Secondary end points were achievement of complete remission (CR), CR with incomplete peripheral count recovery (CRi), relapse-free survival, relapse and death in remission (the last three for patients achieving either CR or CRi), together with resource use and toxicity (haematologic recovery times and non-haematologic toxicity scored using National Cancer Institute Common Toxicity Criteria, Version 3).

All end points were defined according to the revised International Working Group criteria,^16^ including the use of competing-risks methodology to estimate cumulative incidence of relapse (CIR), with the exception that in the protocol as written, the definition of CR did not require count recovery; however, because these data were routinely available, in this report, we divided patients into those who achieved CR by International Working Group criteria and those who instead achieved CRi. On the recommendation of the Data Monitoring and Ethics Committee, the trial was analyzed once a minimum of 1-year follow-up was available and a minimum of 552 deaths had been observed. Follow-up was complete for 98% of patients to 1 March 2015; follow-up for patients was censored on the date they were last known to be alive. Median follow-up was calculated using reverse censoring. Survival for patients withdrawing from follow-up was censored on the date of withdrawal; one patient withdrew consent in this cohort. All analyses were performed by intention to treat; time-to-event data were summarised using Kaplan–Meier or cumulative incidence estimates and analyzed using the log-rank test, with the Mantel–Haenszel test used for dichotomous outcomes such as remission. Resource usage and toxicity data were compared using the Wilcoxon rank-sum test. As per the statistical analysis plan, analyses of outcome were performed, stratified by the randomisation stratification parameters (age, WBC, WHO performance status, type of disease (*de novo*, secondary, high-risk MDS), and entry into the other induction randomisation) as well as the cytogenetic group (defined using the MRC classification^14^) and other potentially important factors, such as *FLT3-ITD* and *NPM1* mutations. Such stratified analyses are presented in Forest plots using standard meta-analytic techniques, with suitable tests for interaction (trend or heterogeneity) performed. Because the trial was designed factorially, later randomisations did not affect the results of the induction randomisations, because allocation was stratified for treatment received in induction.

The results of the gemtuzumab ozogamicin randomisation have already been reported in full,^17^ and the consolidation and maintenance randomisations will be reported elsewhere.

Minimal residual disease assessment by flow cytometry, with a sensitivity of 1 × 10^4^, was undertaken in 135 of 410 randomised patients who were in morphological remission after the first induction course. The methods have been described in detail elsewhere.^18^ Samples of bone marrow were collected after recovery from 135 patients in CR post course and 154 patients in CR post course 2.

## Results

Between August 2006 and December 2008, 806 untreated patients from 124 centres in the UK, Denmark and New Zealand were randomised to receive either two induction courses of DClo: daunorubicin 50 mg/m^2^ on days 1, 3 and 5 combined with clofarabine 20 mg/m^2^ on days 1–5, or two courses of DA: daunorubicin 50 mg/m^2^ combined with ara-C 100 mg/m^2^ b.i.d. days 1–10 in course 1 and days 1–8 in course 2. Fifty-nine patients were excluded from the randomisation based on renal criteria and were allocated to the daunorubicin/ara-C induction, but were eligible to be considered for the other trial randomisations.

**Figure 2:**
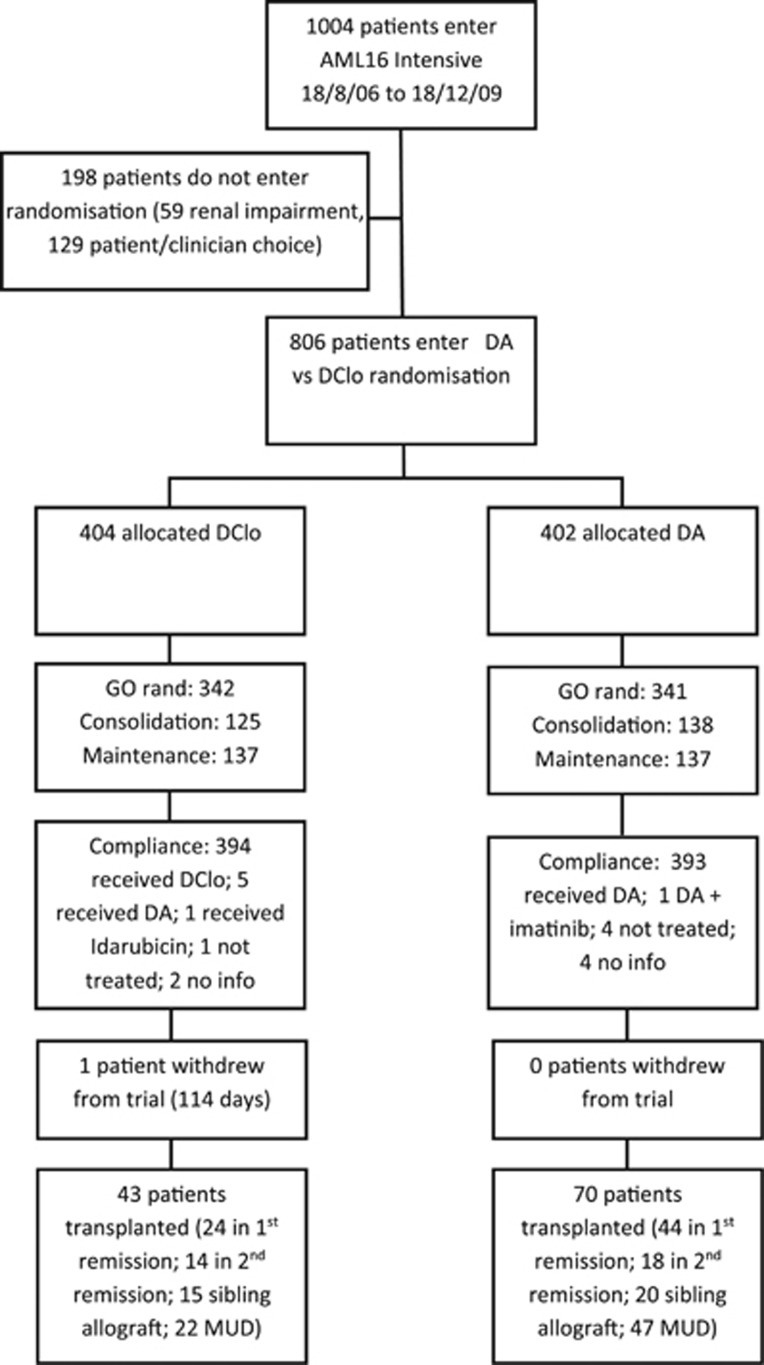
CONSORT diagram.

The other interventions included gemtuzumab ozogamicin 3 mg/m^2^ or not in course 1. Patients who achieved a complete remission (CR), complete remission with incomplete platelet recover (CRi), or a partial remission (<15% blasts in the bone marrow) in response to course 1 and were in CR after 2 induction courses were eligible to be randomised to have a single consolidation course (daunorubicin 50 mg/m^2^ on days 1 and 3 combined with ara-C 100 mg/m^2^ b.i.d. on days 1–5) or no consolidation, and to receive, or not receive, maintenance treatment with azacitidine 75 mg/m^2^ for 5 days every 6 weeks for 12 months (9 courses). The disposition of patients is shown in the CONSORT diagram (Figure 2).

The compliance with the allocated treatment was 98% and the overall response rate (CR+CRi) was 68% and survival at 5 years is 15%.

### Remission induction

The results of induction treatment are summarised in Table 2. Overall, 61% of patients entered CR, and an additional 7% achieved marrow remission with incomplete recovery of peripheral blood counts (i.e. CRi). Remission (CR or CRi) was recorded after course one in 51% of patients; after course two, in an additional 14%. In 3% of patients remission took more than two courses. There was a trend for a superior CR rate in the DA patients 64% vs DClo, 58% (OR 1.30 (0.98–1.73), *P*=0.07), and an additional 6 and 8%, respectively, achieved a CRi; thus, the overall response rate was 71% vs 66% (HR 1.26 (0.94–1.70), *P*=0.12). The induction deaths were not different (11% vs 11% OR 0.99 (0.64–1.55), *P*=1.0) and 30-day (9% vs 8%) and 60-day (15% vs 14%) mortalities were not significantly different (Table 2).

In the samples in morphological remission which were assessed for MRD, the positivity after course 1 in the DA patients was 52% and in the DClo patients was 54% (*P*=0.9). After course 2 the MRD positivity was 34 and 39%, respectively (*P*=0.5). There was no suggestion of a benefit of any particular demographic or cytogenetic subgroup, whether an *FLT3* or *NPM1c* mutation as present or whether the patient received gemtuzumab ozogamicin or not (Supplementary Figure S1).

### Toxicity

With the exception of diarrhoea (12% with DA in course 1) and nausea (10% with DClo in course 1), no grade 3 or 4 toxicity was recorded in more than 10% of patients on either treatment arm during either induction courses (Table 3). The median day of recovery of neutrophils and platelets measured from the end of the course was significantly longer in the DClo arm (neutrophils 20 vs 24 days, *P*<0.0001; platelets 21 vs 24 days, *P*<0.0001). Significantly more red cell and platelet transfusions, days on antibiotic and days in hospital were required after course 1 in the DA arm (Table 3). There was little difference in transfusion requirement after course 2.

### Relapse

**Figure 3:**
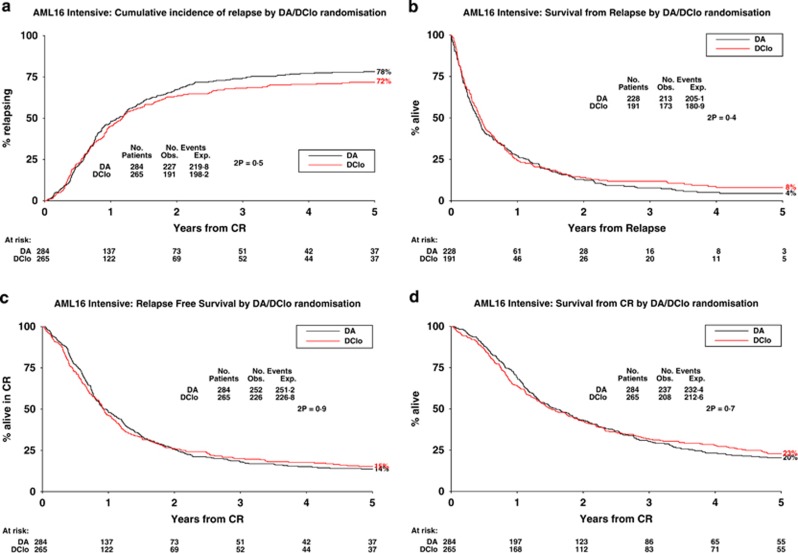
Outcomes. (**a**) Cumulative incidence of relapse; (**b**) survival post relapse; (**c**) relapse-free survival; and (**d**) survival from CR.

The five-year cumulative incidence of relapse (Figure 3a) was 75% (DA 78% vs DClo 72% HR 0.93 (0.77–1.13), *P*=0.5). In the exploratory analysis of subgroups no differences were found (Supplementary Figure S2). The respective survival from relapse (Figure 3b) was also not different (4% vs 8% HR 0.92 (0.75–1.13), *P*=0.4).

### Relapse-free survival

Death without relapse was nonsignificantly increased in the DClo arm (8% vs 13% HR 1.47 (0.89–2.43), *P*=0.13). Taken together with the nonsignificant difference in relapse, no difference was found between the arms in relapse-free survival (14% vs 15%) (HR 0.99 (0.83–1.19), *P*=0.9; Figure 3c) or survival from CR (20% vs 23% HR 0.96 (0.80–1.16), *P*=0.7; Figure 3d).

### Overall survival

**Figure 4:**
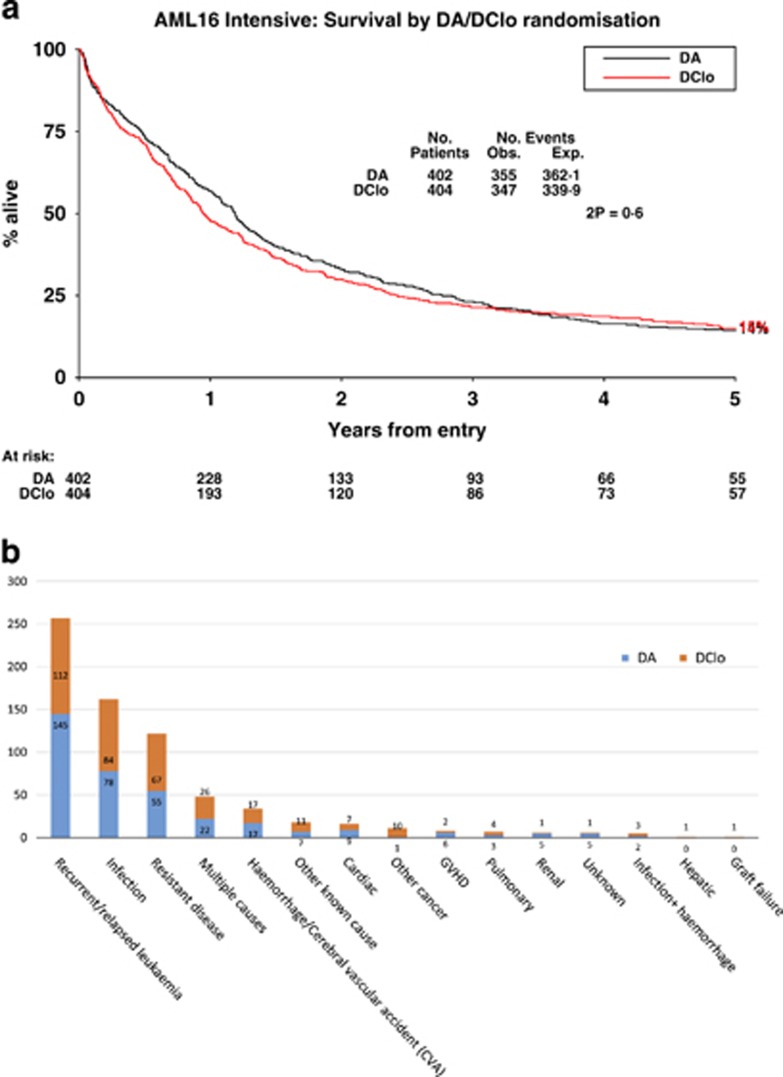
Overall survival: (**a**) survival by arm and (**b**) histogram of causes of death.

No difference was found in survival (DA 15%, DClo 14% HR 0.96 (0.67–1.39), *P*=0.8; Figure 4a) either overall or within exploratory subgroups (Supplementary Figure S3). In particular, there was no significant interaction with any other treatments. There were no obvious differences between the causes of the 355 deaths on the DA arm vs the 347 on the DClo arm with about half in each case being due to refractory or recurrent disease (Figure 4b).

## Discussion

The inability to develop an improved induction regimen for older patients with AML is a major issue, but it is also true for younger patients, such that the combination of daunorubicin and ara-C remains the standard of care. Variations in dose and scheduling of the two drugs have been explored in many studies in patients of all ages, but dose escalation options are limited in older patients.^19, 20, 21, 22, 23^ The general use of the higher dose (90 mg/m^2^) of daunorubicin was not of overall benefit in older patients although it did improve outcome in the subset aged 60–65 years when compared with a 45 mg/m^2^ dose level.^20^ Gemtuzumab ozogamicin in this NCRI AML16 trial when added to induction course 1 in a small dose of 3 mg/m^2^ produced an OS benefit, as it did in the French ALFA study in patients aged 50–70 years using a different schedule.^17, 24^ In our AML15 trial, which was intended for younger patients (<60 years), we observed a powerful antileukaemic effect when using the FLAG-Ida (fludarabine/ara-C/G-CSF/Idarubicin) combination,^25^ which is now being prospectively assessed in older patients.

Alternative nucleoside analogues have recently been explored in AML with mixed results. The addition of cladrabine^26^ to standard DA treatment appears promising particularly in younger patients with adverse risk cytogenetics. Single-agent sapacitabine in older unfit patients was not beneficial,^27^ but other studies in combination are underway or yet to be reported. Clofarabine as monotherapy has been rather ambitiously compared with standard 3+7 daunorubicin, but was unsuccessful.^28^ An obvious setting in which to assess clofarabine was as a replacement for ara-C in combination with an anthracycline. This had a number of attractions. First it had been developed as a 5-day schedule compared with the need for 7 or 10 days of ara-C. Second, in the preliminary studies it appeared to be equally effective in adverse risk patients who of course are well represented in an older population. Third, it had the prospect of being taken orally.

In the initial studies in relapsed disease undertaken by the MD Anderson group,^10^ the dose used was 40 mg/m^2^. However when initially targeting the older unfit patient population we felt that a lower dose of 30 mg/m^2^ would be wise. Even at this dose in unselected older patients some renal toxicity was observed, which required more rigorous entry criteria with respect to renal function. We also explored a lower daily dose of 20 mg/m^2^,^11^ which was found to be well tolerated, with less renal and hepatic biochemical abnormalities being seen, without any loss of efficacy. This dose level was then explored as monotherapy in older patients judged to be unfit for conventional therapy.^11, 12^ After a brief safety assessment of the combination with daunorubicin in older patients considered suitable candidates for intensive therapy,^29^ the randomised comparison was initiated. The results presented here provide no evidence overall, or in any subgroup, to suggest that ara-C should be replaced by clofarabine, at least at the 20 mg/m^2^ daily dose. Taken together, and acknowledging the issue of exploring higher doses in combination, the clinical experience so far does not suggest that clofarabine should displace existing treatments. New treatment options such as addition of inhibitors of *FLT3, IDH1* and *IDH2* hold promise, but also challenges because of the lower frequency of specific mutations in older patients. Of recent interest is the liposomal formulation of the combination of daunorubicin and ara-C (CPX-351) which is a novel way of delivering therapy and has proved to be more effective in older patients with secondary AML.^30, 31^

## Figures and Tables

**Table 1:** Patient characteristics by arm

*Characteristics*	*DA (*n=*402)*	*DClo (*n=*404)*
*Age*		
<60	8	8
60–64	115	116
65–69	168	168
70–74	89	89
75+	22	23
Median (range)	67 (56–84)	67 (56–80)

*Sex*		
* *Female	172	154
Male	230	250

*Diagnosis*		
*De novo*	289	290
Secondary	69	71
High-risk MDS	44	43

*WBC (× 10*^*9*^*/l)*		
<10	249	251
10–49.9	112	111
50–99.9	23	25
100+	18	17
Median	4.1 (0.2–266.0)	5.2 (0.2–336.7)

*Performance status*		
WHO PS 0	250	250
WHO PS 1	130	130
WHO PS 2	13	16
WHO PS 3,4	9	8

*Cytogenetics*		
Favourable	15	9
Intermediate	229	228
Adverse	67	77
Unknown	91	90

*Wheatley group*		
Good	129	112
Standard	137	141
Poor	136	151

*FLT3-ITD*		
Wild type	101	97
Mutant	14	18
Unknown	287	289

*NPM1*		
Wild type	92	80
Mutant	19	23
Unknown	291	301

*GO induction*		
GO	171	172
No GO	170	170
Not randomised	61	64

*Consolidation*		
2 courses	68	63
3 courses	70	62
Not randomised	264	279

*Maintenance*		
Maintenance	67	70
No maintenance	70	67
Not randomised	265	267

Abbreviation: GO, gemtuzumab ozogamicin.

**Table 2:** Outcomes by treatment arms—all estimates are at 5 years except if stated otherwise

	*DA (%)*	*DClo (%)*	*HR/OR, 95% CI*	P*-value*
CR	64	58	1.30 (0.98–1.73)	0.07
CRi	6	8		
ORR (CR+CRi)	71	66	1.26 (0.94–1.70)	0.12
Resistant disease	18	24	1.36 (0.97–1.91)	0.08
Induction death	11	11	0.99 (0.64–1.55)	1.0
30-day mortality	9	8	0.98 (0.61–1.57)	0.9
60-day mortality	15	14	0.96 (0.67–1.39)	0.8
Overall survival	14	15	1.04 (0.90–1.21)	0.6
Relapse-free survival	14	15	0.99 (0.83–1.19)	0.9
Cumulative incidence of relapse	78	72	0.93 (0.77–1.13)	0.5
Cumulative incidence of death in CR	8	13	1.47 (0.89–2.43)	0.13
Survival post CR	20	23	0.96 (0.80–1.16)	0.7
Survival post relapse	4	8	0.92 (0.75–1.13)	0.4

**Table 3:** Toxicity outcomes—tests are by Wilcoxon rank-sum test except for *(log-rank test)

*Toxicity*	*DA*		*DClo*		P*-value*
	*% grade 3–4*	*Mean grade*	*% grade 3–4*	*Mean grade*	
*Course 1*					
* *Nausea	5%	0.8	10%	1.0	0.01
* *Oral	3%	0.7	2%	0.7	0.4
* *Diarrhoea	12%	1.1	8%	1.0	0.12
* *Cardiac	7%	0.4	5%	0.3	0.3
* *Liver AST	6%	0.6	6%	0.5	0.6
* *Liver ALT	4%	0.6	6%	0.7	0.3
* *Bilirubin	7%	0.8	5%	0.7	0.04
* *Median days to neutrophil recovery	20		24		<0.0001*
* *Median days to platelet recovery	21		24		<0.0001*
* *Mean blood units	11.0		9.5		0.0002
* *Mean platelet units	12.9		9.8		<0.0001
* *Mean days antibiotics	19.7		16.7		<0.0001
* *Mean hospitalisation days	33.8		31.4		<0.0001

*Course 2*					
* *Nausea	4%	0.7	7%	0.9	0.004
* *Oral	1%	0.4	1%	0.4	0.9
* *Diarrhoea	4%	0.6	9%	0.7	0.19
* *Cardiac	2%	0.1	2%	0.2	0.7
* *Liver AST	1%	0.2	4%	0.4	0.06
* *Liver ALT	3%	0.4	2%	0.6	0.004
* *Bilirubin	3%	0.5	5%	0.4	0.4
* *Median days to neutrophil recovery	20		21		0.2*
* *Median days to platelet recovery	25		24		0.3*
* *Mean blood units	6.3		6.4		0.9
* *Mean platelet units	6.0		5.7		0.03
* *Mean days antibiotics	9.4		9.6		0.6
* *Mean hospitalisation days	25.0		23.4		0.01
