# Supplementary material for: A comparison of clofarabine with ara-C, each in combination with daunorubicin as induction treatment in older patients with acute myeloid leukaemia
Source: Leukemia. 2016 Sep 30;31(2):310–7. doi: 10.1038/leu.2016.225 (PMC5292678; doi:10.1038/leu.2016.225)

**Supplementary Figure 1: Stratified analysis of CR**


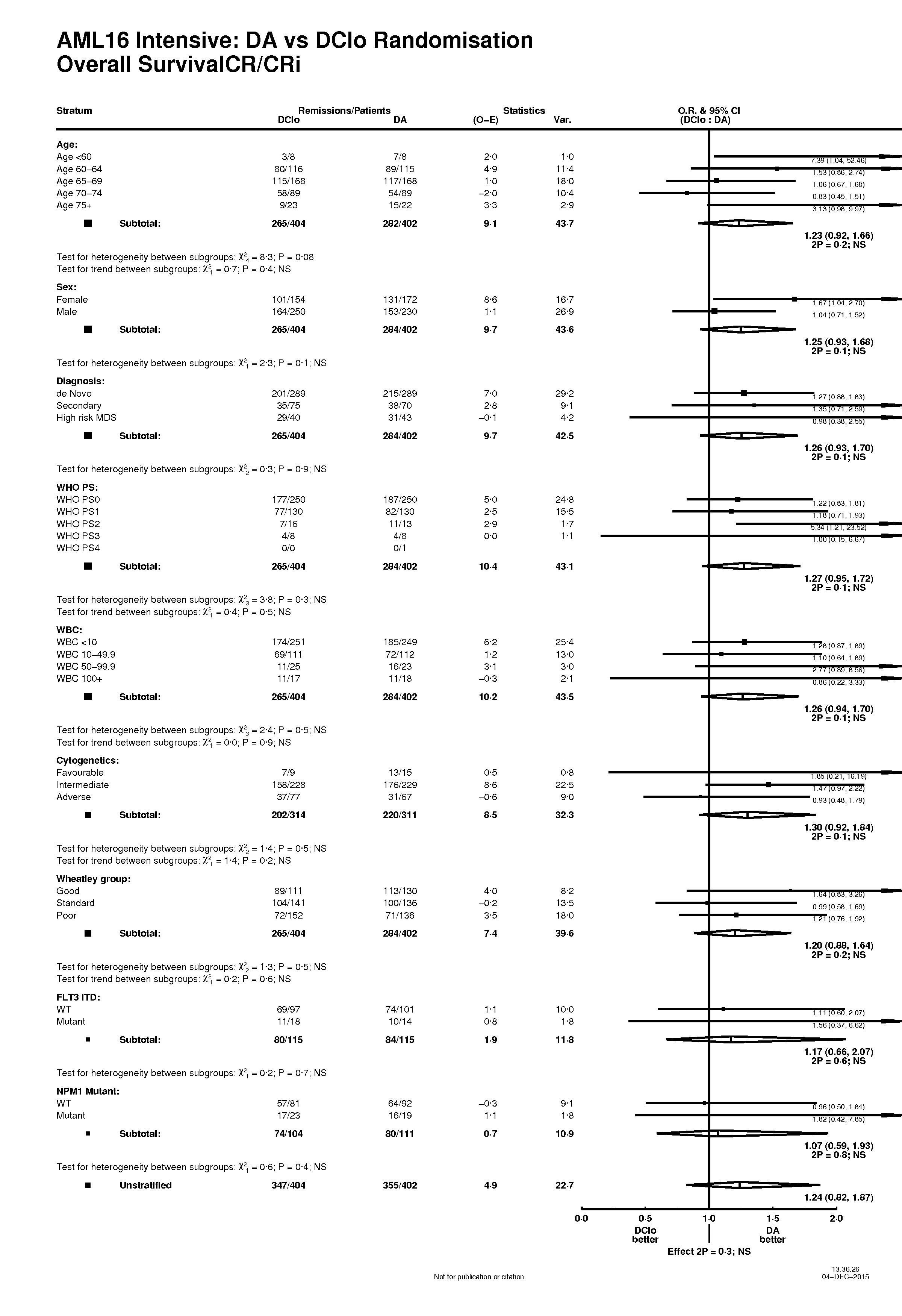


**Supplementary Figure 2: Stratified analysis of relapse**


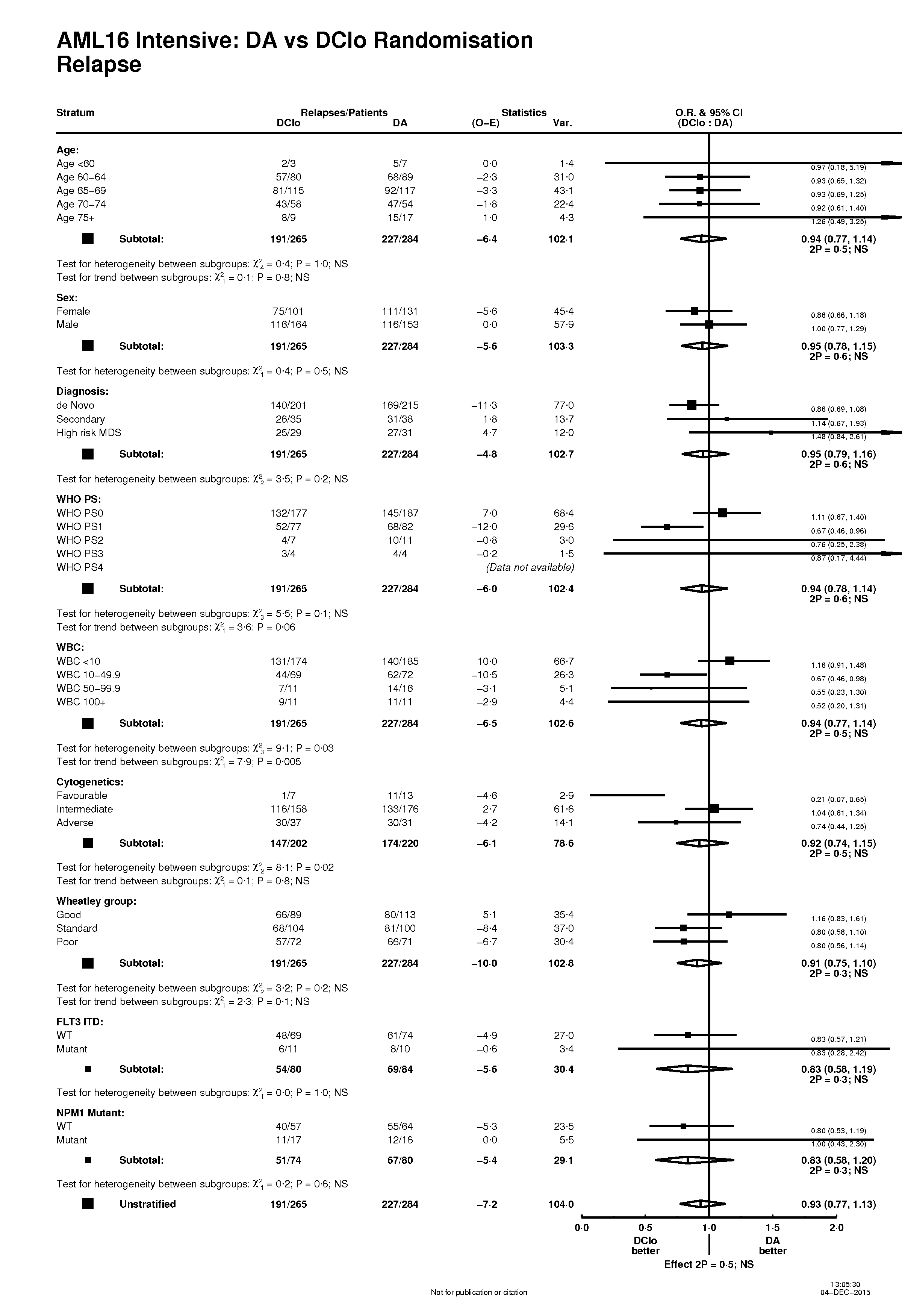


**Supplementary figure 3: Stratified analysis of overall survival**


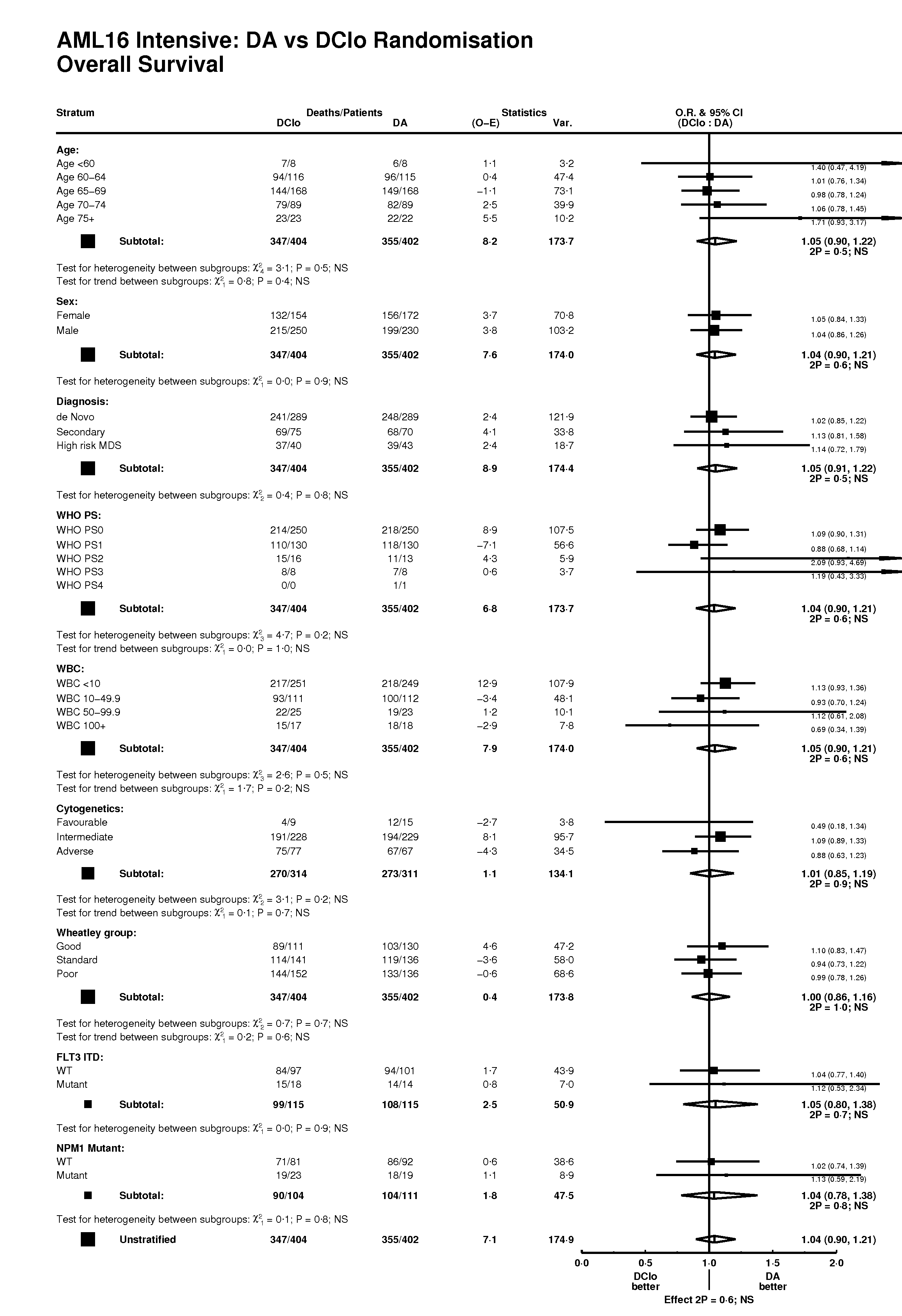

Supplement: Supplementary Figures [file leu2016225x1.docx]
